# Smilebright^RO^—Study Protocol for a Randomized Clinical Trial to Evaluate Oral Health Interventions in Children

**DOI:** 10.3390/mps8030049

**Published:** 2025-05-07

**Authors:** Ruxandra Sava-Rosianu, Guglielmo Campus, Vlad Tiberiu Alexa, Octavia Balean, Ruxandra Sfeatcu, Alice Murariu, Alexandrina Muntean, Daniela Esian, Constantin Daguci, Simona Olaru-Posiar, Vanessa Bolchis, Antonia Ilin, Ramona Dumitrescu, Berivan Laura Rebeca Buzatu, Mariana Postolache, Nicoleta Toderas, Roxana Oancea, Daniela Jumanca, Atena Galuscan

**Affiliations:** 1 Translational and Experimental Research Center in Oral Health, “Victor Babes” University of Medicine and Pharmacy Timișoara, 300040 Timișoara, Romania; sava-rosianu.ruxandra@umft.ro (R.S.-R.); vlad.alexa@umft.ro (V.T.A.); vanessa.bolchis@umft.ro (V.B.); antonia.sabo-meze@umft.ro (A.I.); berivan.buzatu@umft.ro (B.L.R.B.); roancea@umft.ro (R.O.); jumanca.daniela@umft.ro (D.J.); galuscan.atena@umft.ro (A.G.); 2 Department of Cariology, Institute of Odontology at Sahlgrenska Academin, University of Gothenburg, 40530 Gothenburg, Sweden; guglielmo.giuseppe.campus@gu.se; 3 Department of Dental Sciences and Maxillo-Facial Surgery, Sapienza University of Rome, 00185 Rome, Italy; 4 Department of Cariology, Saveetha Dental College and Hospitals, SIMATS, Chennai 600077, India; 5 Department of Oral Health and Community Dentistry, Faculty of Dentistry, “Carol Davila” University of Medicine and Pharmacy, 041292 Bucharest, Romania; ruxandra.sfeatcu@umfcd.ro; 6 Department of Community Dentistry, Faculty of Dental Medicine, University of Medicine and Pharmacy “Grigore T. Popa”, 700115 Iasi, Romania; alice.murariu@umfiasi.ro; 7 Department of Pedodontics, Faculty of Dental Medicine, University of Medicine and Pharmacy “Iuliu Hatieganu”, 400083 Cluj-Napoca, Romania; alexandrina.muntean@umfcluj.ro; 8 Department of Pedodontics, Faculty of Dental Medicine, University of Medicine and Pharmacy, 540139 Targu Mures, Romania; daniela.esian@umfst.ro; 9 Department of Oro-Dental Prevention, Faculty of Dental Medicine, University of Medicine and Pharmacy of Craiova, 200349 Craiova, Romania; dagucicristi@yahoo.com; 10 Faculty of General Medicine, “Victor Babes” University of Medicine and Pharmacy Timișoara, 300040 Timișoara, Romania; olaru-posiar.simona@umft.ro; 11 Department of Public Health, University of Medicine and Pharmacy Bucharest, 041292 Bucharest, Romania; postolache_mariana@yahoo.com; 12 Specialization in Clinical Psychology and Psychotherapy, Department of Psychology, Faculty of Sociology and Psychology, West University of Timișoara, 300223 Timișoara, Romania; nicoleta.toderas01@e-uvt.ro

**Keywords:** oral health, education, children, nursery, dental hygienist

## Abstract

Background: Oral diseases represent a constant burden for health care and socio-economic systems as they are correlated to other non-communicable diseases. The aim of the proposed intervention is to test the effect of daily tooth brushing and oral health education on the oral health status of kindergarten children. Methods: The protocol will be conducted based on a previous epidemiological survey and conducted over 24 months; it has been developed on different levels. Dental hygienists will receive specific training to deliver oral health promotion to children and nursery educators. Training will focus on tailoring key messages to the specific age at visit; this will be outlined in the care pathway and offer practical preparation for delivering interventions and a toothpaste/toothbrush scheme. It will also, involving involve offering free daily tooth brushing to every 4–6-year-old child attending nursery. Data will be collected in four kindergartens in the capital or metropolitan areas, two kindergartens each in two large cities, and one kindergarten each in four villages from different geographic areas. Procedures used to assess the outcomes of each activity will be tailored to specific outcomes. Daily tooth-brushing activities will be monitored using qualitative research. A cost analysis including the distribution of necessary materials and correct delivery of products that shows price trends and percentage differences over the time span as well as consumer price index evaluation for the given time span will be conducted. Clinical outcomes will be evaluated using the caries incidence rate; this will be calculated for each tooth as the unit of analysis and evaluated using a multi-step approach. Discussion: Downstream oral health prevention interventions, like clinical prevention and oral health promotion, aim to enhance children’s quality of life. The program’s goal is to progress towards upstream interventions for a more significant impact.

## 1. Introduction

The 74th World Health Assembly recognized oral diseases as strictly correlated to other non-communicable diseases. Oral diseases represent a constant burden for both health care and socio-economic systems [1]. Dental caries and periodontal disease are the major oral diseases worldwide. In children under the age of 6, the disease, called Early Childhood Caries (ECC), occurs in children under the age of 6 and has a specific pattern that leads to significant modifications in the child’s functional development [2]. The disease is, nowadays, considered reversible if it is diagnosed in the early, non-cavitated stage, and can be halted even if indicators suggest microcavitation of the enamel. However, it remains the most common chronic disease in young children, and it has also been associated with poor quality of life for both affected children and their parents, affecting their ability to learn, causing missed school days, and incurring high costs for treatments [3]. The presence of ECC in very early childhood is linked to a higher risk of developing new caries lesions in the permanent dentition and an increased number of emergency dental visits due to caries complications [4].

Good behaviors and skills gained during early childhood are crucial for maintaining good oral health throughout life [4,5]. In addition to oral hygiene, there are several other factors that influence oral health status, such as attending dental visits, socio-economic status, or parental dental attendance, especially maternal attendance [4,5,6]. During adult life, healthy hygiene behaviors such as tooth brushing and additional dental hygiene measures are correlated to dental visits, dental plaque accumulation, the presence of calculus, and dental anxiety [7].

The scientific literature provides extensive evidence of oral health inequalities due to specific oral health behaviors, such as dietary habits, hygiene practices, bad habits, and dental check-ups [6,8,9]. This dynamic process, which has been extensively studied using the life course framework, involves a number of variables. Oral health status was linked to several covariates, such as socio-economic status, maternal dental visit behavior, and local risk factors, such as the presence of plaque, calculus, dental anxiety, and oral hygiene patterns, such as tooth-brushing frequency and use of additional hygiene products [8]. In addition, lower attendance to dental care services can be seen among younger children, edentulous patients, patients who suffer severe tooth loss, and have poor health literacy, as well as those with general and oral health issues [9]. However, these findings are mainly cross-sectional rather than longitudinal. Oral health interventions are often not a priority for medical staff and health policy developers in several countries, with other diseases considered more important. Another issue is that there are insufficient evidence-based interventions that address preventive dental care and oral health risk factors [10,11].

The effectiveness of oral health interventions should focus on the fact that even simple interventions can be influenced by multiple individual factors, including social determinants and health systems. Therefore, they should be considered complex interventions [12].

The evaluation of complex oral health interventions is often difficult because of the complex mechanisms involved in the processes. Therefore, rather than attempting to evaluate the effectiveness of the intervention without taking into account all the covariates, research should focus more on evaluating methods of implementation, appropriate target populations, and requirements that need to be met before application. To implement an intervention, details regarding feasibility, steps, and working components should be first considered.

Several guidelines and frameworks for the development, implementation, and evaluation of health interventions have been published by other countries like Scotland (“Childsmile”), Wales (“Designed to smile”), Brazil (“Brazil Sorridente”) [13,14,15], but data on preventive oral health interventions are still scarce. Thus, the design proposed for this intervention focuses on the details of the development and implementation of an oral health intervention for young children.

### 1.1. Objectives

The null hypothesis is that daily tooth brushing and oral health education in kindergartens have no effect on the caries status of children aged 4-6. To test the hypothesis, the project will aim to establish the association between those variables. Ancillary objectives will be to improve oral health and reduce inequalities in both dental health and access to dental services by shifting the balance of care towards more preventive and anticipatory care and promoting health improvement.

### 1.2. Expected Outcome

The clinical relevance of the findings is that preventive care includes good oral health behaviors such as low sugar consumption, health education, and daily oral hygiene using a toothbrush and fluoride toothpaste, as well as additional aids such as interdental brushes, dental floss, or mouthwashes that can help remove plaque and disrupt biofilm.

## 2. Experimental Design

The study is designed and planned as an RCT, taking into consideration the early diagnosis of caries lesions through clinical examinations in the nurseries, control of risk factors through the evaluation of oral health-related knowledge, and behaviors, health education, and evaluation of the efficacy to arrest caries’ progression through the application of fluoride toothpaste. The trial will also develop new primary care teams—dental hygienists. This will provide a framework for integrating the prevention and control of dental caries into general health interventions.

In accordance with the informed consent, all personal data of the participants will be kept anonymous. The participants will receive a unique ID which will be used for data entry on an online software. The ID will be used for each clinical examination and data will be entered accordingly. As stated by Romanian regulations, an archive of the data has to be kept for 5 years after the trial in the secured archive of the university. The program was approved and registered by the Ethical Committee of the “Victor Babes” University of Medicine and Pharmacy Timișoara, Romania, under trial No. 18688/23.10.2023 and Protocol NCT06441500/28.05.2024 released on https://clinicaltrials.gov/, accesed on 29 May 2024, protocol version 1.

The project will be implemented in collaboration with the universities of Medicine and Pharmacy in Bucharest, Timișoara, Cluj-Napoca, Craiova, Iasi, and Targu Mures in order to ensure a homogeneous distribution of the evaluation sites, covering the whole country. The study group will consist of kindergarten children. The pathfinder sampling technique will be used to obtain a representative sample. Selection of nurseries will be performed according to the pathfinder sampling technique. As suggested by the World Health Organization (WHO), this survey’s design is suitable for the collection of data for planning purposes and monitoring of oral health programs in all countries regardless of the level of disease, availability of resources, or complexity of care.

**Figure 1:**
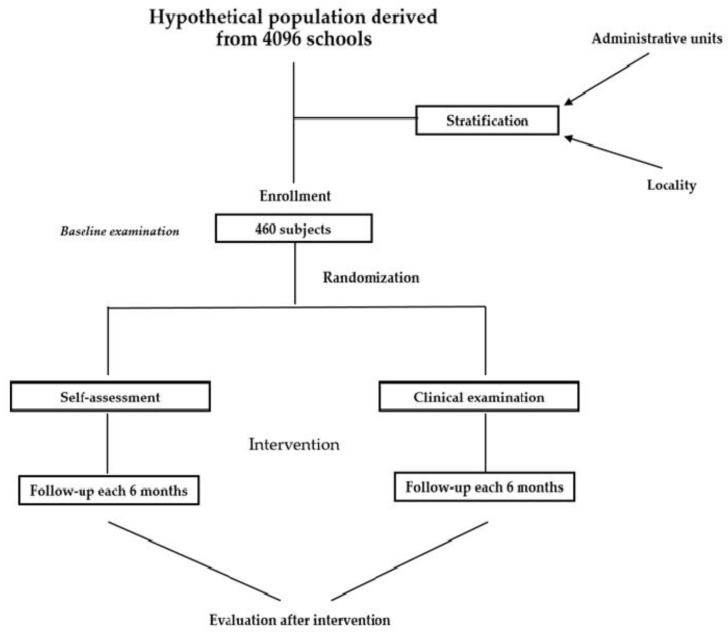
RCT design flowchart.

The number of children is based on an initial calculated requirement of 415 participants, with an additional 10% (rounded up from 456.5 to 460) added to account for potential dropouts or loss to follow-up. The sample is stratified based on administrative units and locality type and randomization is ensured by using the MS Excel function. Children will be included based on the enrolment in kindergarten, consent of the parents, and assent from the child. The exclusion criteria are represented by chronic general diseases or a history of allergic reactions, the presence of fluorosis or other enamel deficiencies, and non-collaborative behavior. The selection can be seen in the flowchart in Figure 1.

The nationally representative sample will be evaluated at baseline using self-assessment tools and clinical examination. During the intervention, evaluations will be performed every 6 months.

**Figure 2:**
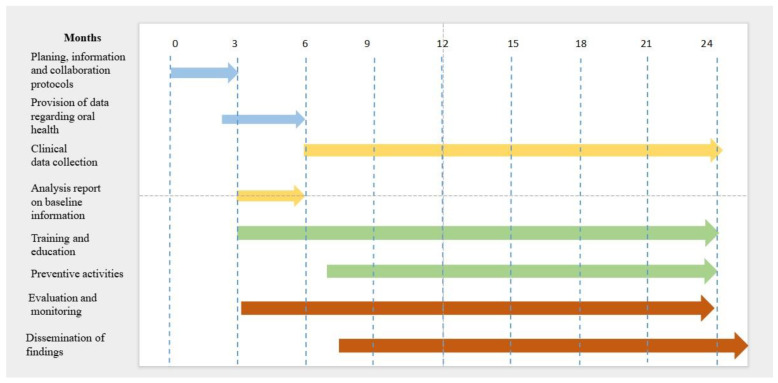
Activity flowchart.

The program is designed to deliver additional clinical prevention activities through dental hygienists, aimed at children aged four to six, a special priority being afforded to nursery establishments, in order to improve the oral health of young children and complement the established national toothpaste/toothbrush scheme. The activity flow chart can be seen in the figure below (Figure 2).

### Study Setting

**Figure 3:**
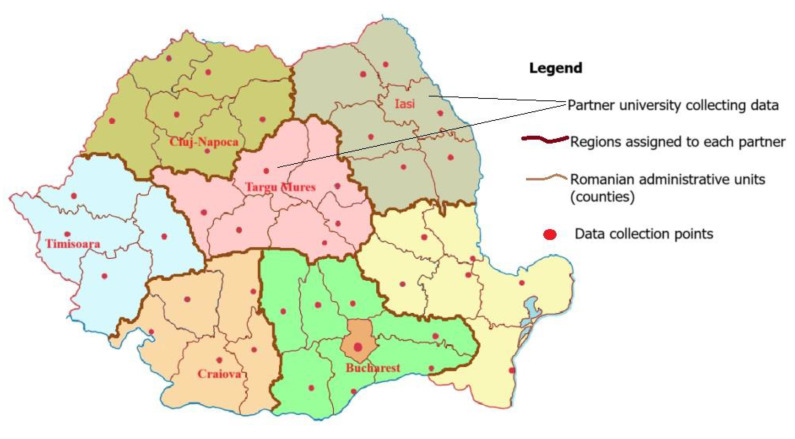
Distribution of data collection points.

The total sample size is calculated according to the WHO guidelines using a stratified sampling technique for examination sites in Romanian cities: Bucharest, Timișoara, Cluj-Napoca, Craiova, Iasi, Targu Mures, and the surrounding geographical areas. Data collection points (Figure 3) will be 4 kindergartens in the Capital or metropolitan areas, 2 kindergartens in each of 2 large cities, and 1 kindergarten in each 4 villages from different geographic areas (Dobrogea, Moldova, Ardeal, Muntenia). Metropolitan areas in Romania are Iași, Brașov, Oradea, Târgu Mureș, Cluj Napoca, Craiova, Roman, Baia Mare, Satu Mare, Timișoara and Arad. Romania is divided into 40 counties (regional administrative sites consisting of several cities and villages), the capital, Bucharest being considered on its own. Further on, it is divided into metropolitan areas (over 250,000 inhabitants), large cities (100,000–250,000 inhabitants), small cities (less than 100,000 inhabitants), and villages (less than 5000 inhabitants). A detailed list of the study sites can be obtained from the project manager.

Children aged 4–6 years, enrolled in kindergartens in Romania will be included in the study, providing they show signed informed consent from their parents/caregivers. Parents or caregivers are responsible for signing the consent form for children and any subject may withdraw from the study at any time after recruitment. The examiners shall collect the informed consent forms prior to the examination. Before each examination, each subject will have the free will to participate by direct inquiry.

## 3. Procedure

Pre-intervention baseline data will be collected according to the WHO stepwise approach—self-assessment and collection of clinical data will be used to plan and evaluate further health interventions. Self-assessment information on children’s oral health behaviors, like diet, frequency of brushing, use of fluoridated toothpaste, use of additional oral hygiene aids (interdental brushes and mouthwash), frequency of dental check-ups, and reason for last dental visit, will be collected using the previously developed National Oral Health Survey questionnaire [15]. Answers will be obtained from parents/caretakers before clinical examination. In the unlikely case that no family member or tutor will be able to read the questions, they will be assisted by the educators. The working tool consists of 15 items that make up the two dimensions of types of behavior (prevention and nutrition) and family characteristics (parent’s level of education and working status). The two behaviors will be correlated with clinical indices that will help to delimit the existence or lack of certain significant differences among children. In a concrete way, for a better understanding of the risk factors, the scientific approach aims to relate to not only specific elements of the child (gender, age, parental education, and environment of residence), but also elements related to the objective reality in which the child lives (development index and development region of the county where the child comes from, type of city, etc.). Reexamination will be performed twice a year to ensure data comparison.

Clinical examinations will be performed using the International Caries Detection and Assessment System (ICDAS) criteria by a calibrated medical staff from each partner‘s university. ICDAS represents a caries detection and assessment system that quantifies caries lesions into 6 categories: code 1 represents demineralized lesions (white spot lesions) which can be only seen after air-drying the surface for 5 s; code 2 are demineralized lesions that can be observed without air-drying; code 3 are micro-cavities in enamel; 4 are lesions in dentin, observed as a dentinal shadow; and 5 and 6 are already cavitated lesions which affect less or more than half of the tooth surface. Calibration will be performed every six months according to the ICCMS (International Caries Classification and Management System) guidelines calculating the intra- and inter-examiner correlation coefficients (kappa-index). The level of agreement has to be more than 80%. Children will be examined in a seated position, using a dental mirror and a probe, without air-drying. Thus, ICDAS codes 1 and 2 will be counted together as “A”.

Volunteer dental hygiene students will be trained to deliver oral health promotion to nursery educators and oral health promotion activities for children, focusing on the tailoring of key messages to the specific age and on practical preparation for delivering interventions in nurseries along with implementing the toothpaste/toothbrush scheme involving free daily tooth brushing to every 4 and 6-years old child attending nursery. The Bass brushing technique will be taught to the children and they will brush their teeth once a day while present in the kindergarten. The technique has been chosen because it was proven to be the most effective method in young children [16,17,18]. Fluoridated toothpaste will have a concentration of 1000 ppm Amin fluoride.

Children will receive educational lessons using PowerPoint presentations on a regular basis. Educators will be provided with brochures containing information on the tooth-brushing technique as well as oral health education: healthy diet, proper oral hygiene, frequency of dental check-ups, etc.

A detailed participant timeline is presented in Table 1.

### 3.1. Strategies to Improve Adherence to Interventions

Adherence to intervention protocols will be constantly monitored and evaluated. Evaluation of the intervention will focus on three main domains as follows:

1. Specific training for dental hygienists to deliver oral health promotion to children, parents, and nursery staff, focusing on tailoring key messages to the specific age at visit outlined in the care pathway, and on practical preparation for delivering interventions in the nurseries. All the procedures used to assess the outcomes of each activity will be tailored to the specific outcomes. Questionnaires will be developed by a team of sociologists and psychologists, pretested, and used before and after the interventions in order to assess possible changes in knowledge level. The questionnaires will be structured into three distinct sections: the first referring to personal characteristics, the second addressing general knowledge concerning both general and oral health, and the third focusing on awareness related to the dissemination of information and educational competencies. The Capacity-Building Framework will be used to link knowledge to actions that test the skills acquired.
2. A toothpaste/toothbrush scheme involving free daily tooth brushing will be established for each 4- and 6-year-old child attending nursery, along with the free distribution of toothpaste/toothbrush packs to every child on at least 8 occasions (every 3 months). Daily tooth-brushing activities will be monitored by qualitative research, meaning that interviews with the educators will be used to assess the number of children who attend the kindergarten, the number of children who actually perform daily tooth brushing, the use of toothpaste, and any barriers in the implementation of the tooth-brushing scheme. The distribution of necessary materials, correct delivery of products, cost analysis showing price trends and percentage differences over the time span, and consumer price index evaluation for the given time span will be assessed as part of the cost efficiency analysis.
3. Evaluation of clinical outcomes—caries activity levels—after implementing the daily tooth-brushing scheme. The outcomes will offer information regarding the particularities of the clinical investigation for the specific age group and the time necessary for the investigation and intervention.

### 3.2. Sample Size

The sample size calculation results in a sample of 460 children aged 4–5 years, considering the following parameters: a type I error of 0.05 (z 1 − alpha/2  =  1.96), power of 0.9 (z 1 − beta  =  1.28), standard deviation (SD) 2, a design effect of 2, and a drop-out rate of 10%. For the odds ratio, a percentage mean difference between groups was used as suggested by literature when significance is found after intervention usually measuring for caries prevalence or increment, with mixed interventions ranging from fluoride varnish and teaching how to properly brush teeth to motivational techniques and oral health education [16,17,18]. Demographics with respect to the region and age of interest were chosen.

The total sample size was calculated according to the WHO guidelines using a stratified sampling technique for examination sites in Romanian cities: Bucharest, Timișoara, Cluj-Napoca, Craiova, Iasi, Targu Mures, and the surrounding geographical areas. Data collection points will be 4 kindergartens in the Capital or metropolitan areas, 2 kindergartens in each of 2 large cities, and 1 kindergarten in each 4 villages from different geographic areas (Dobrogea, Moldova, Ardeal, and Muntenia). Metropolitan areas in Romania are Iași, Brașov, Oradea, Târgu Mureș, Cluj Napoca, Craiova, Roman, Baia Mare, Satu Mare, Timișoara, and Arad.

Data will be collected from 3 types of residential areas: metropolitan areas (150 evaluations), big cities (150 evaluations), and rural areas (100 evaluations). In metropolitan areas and big cities, 25 children will need to be evaluated in the same kindergarten.

In rural areas, more than one kindergarten can be evaluated until the desired number of evaluations is obtained for the specific region. This principle has been added because kindergarten is not mandatory in Romania and the number of children enrolled in kindergarten in rural areas is quite low in some regions of the country.

### 3.3. Assignment of Interventions

In order to create a nationally representative sample, the public information available on the websites of all County School Inspectorates (41 counties plus the Romanian Capital) was analyzed. This process involved the evaluation of publicly available information from 4696 schools, which aided the understanding of the territorial distribution of the target population.

The national representative sample is stratified and randomized. The stratification was performed at the level of administrative units (counties), and on locality type (i.e., urban versus rural localities). For each county, the total number of pupils and the percentage of children for each of these administrative units were computed. The percentage was used to estimate how many children would have to be included in each county. The number was then divided based on locality type (i.e., urban versus rural), and the final target number of evaluations was obtained. The MS Excel’s randomization function was used for each county to select an urban and a rural nursery.

The steps of the allocation sequence are as follows:

1. The order of the counties is decided—each collection point receives several counties and the research team can decide in which county it would be more convenient.
2. Kindergartens in the respective county are contacted to determine their availability to participate in the study and the number of children from the target population available for evaluation.
3. If the 2 kindergartens that are contacted together have at least 18 children (out of the required 25), data collection can begin in the respective county.

If the 2 kindergartens contacted do not have at least 18 children together, the next county on the list will be selected and the procedure returns to steps 2 and 3.

E-mails will be sent to the correspondence address provided to generate the allocation sequence. Consent in principle and an estimate of the number of children in the target population will be requested. If there is no response within the next 5 working days, the kindergarten will be called in to check that the e-mail was received in good condition (e.g., to obtain a working e-mail address or to see the status of the request).

Once the study has been accepted, the director of the nursery will be contacted by telephone to arrange details of the timing of the evaluation, to select the date on which the evaluation will take place, and to find out (by telephone) the number of classes. Consent forms and questionnaires will be sent by courier at least 7 days before the assessment date and their receipt and distribution to parents checked by phone 3 days after sending by courier. The return rate of the questionnaires will be checked by telephone (how many questionnaires have been returned, how many consent forms have been signed by parents) 2 days before the assessment date.

### 3.4. Data Collection and Management

Data will be collected in order to evaluate the daily tooth-brushing program and the number and attendance of children performing daily tooth brushing, and the daily tooth-brushing activities will be monitored. The number of school days, the fact that on Saturdays and Sundays children will not be present at school, the timepoint of the daily brushing (after the first meal or after lunch), and the hypothesis of morning brushing at home will be considered as variables for data analysis.

To assess oral health promotion activities, self-administered questionnaires (using the Likkert scale) before and after the activities will be used to test the ability of hygienists to pass the information to nursery staff, parents, and children developing a framework to assist in the application of capacity-building principles to public health.

Plans for the assessment and collection of outcome, baseline, and other trial data, including any related processes to promote data quality (e.g., duplicate measurements, training of assessors) and a description of study instruments (e.g., questionnaires, laboratory tests) along with their reliability and validity, will be drafted. Data collection forms can be obtained from the corresponding author.

Feedback from stakeholders on barriers/facilitators across the processes will be obtained and the behavior of children, parents, nursery staff, and dental hygienists will be monitored, using quantitative and qualitative methods to assess parents’ attitudes toward the project’s activities and dental hygienists’ and educators’ perceived barriers to delivering the tasks. Quantitative measures will use questionnaires to assess variables. Interviews with parents, educators, and stakeholders will ensure qualitative data to assess different tasks.

### 3.5. Statistical Methods for Primary and Secondary Outcomes

Caries activity levels after implementing the daily tooth-brushing scheme will be evaluated as the primary outcome. The outcomes will offer information regarding the particularities of the clinical investigation for the specific age group and the time necessary for the investigation and intervention. Clinical outcomes will be evaluated by using the caries incidence rate, calculated on each tooth as the unit of analysis and evaluated using a multi-step approach:

The net caries increment for initial, moderate, and extensive caries severity using ICDAS (Δ-initial, Δ-moderate, and Δ-extensive) will be calculated at each follow-up examination.

Events will be defined as a tooth receiving a lesion or as the sum of the Δ-caries changes in status recorded at the baseline examination, at the interim, and at the last examination. The number of events will be appraised by subtracting the number of caries-free teeth at the last examination from those at baseline.

Data analysis will be performed using SPSS version 24 (IBM Corp., Armonk, NY, USA) and the R software (R Core Team 2023). SPSS will be used for initial data cleaning, descriptive summaries, and basic inferential tests. R will be used for advanced modeling, intention-to-treat (ITT) analyses, and reproducible visualizations.

Data will be cleaned and validated prior to analysis. Descriptive statistics will be computed using continuous variables—mean ± SD (if normal), or median [IQR] (if non-normal) and categorical variables—counts and percentages. Baseline comparability between groups will be assessed but not tested for statistical significance (in accordance with CONSORT guidelines) [19]. Primary outcomes will be analyzed using the intention-to-treat (ITT) principle. The choice of statistical test depends on the outcome type. For continuous outcomes, linear regression adjusting for baseline, if available, will be used. Logistic regressions will be performed for binary outcomes and Poisson or negative binomial regression could be used for count data. For the longitudinal data, linear mixed models or generalized estimating equations (GEE) will be used to account for within-subject correlation. To explore relationships between secondary variables, correlation analysis will be performed.

The non-parametric Mann–Whitney U test will be applied to assess the differences across the mean number of events between groups.

The efficacy of the treatment will be assessed following the indication of the CONSORT consensus guidelines including both PP (Per Protocol) and ITT (Intention to Treat) protocols to calculate the reduction in risk ratio (RR) and the related number needed to treat (NNT) value. An event will be defined as the change in status at tooth level, i.e., the development of a new lesion or the progression of an existing lesion to a more severe stage. Cox Proportional Hazards models will be run to assess the factors associated with caries change in status. Estimates will be reported in the hazard ratio (HR) and their respective 95% confidence interval (95%CI). For all the statistical analyses, the statistical significance will be set at α < 0.05.

The extent and pattern of missing data will be explored. If data are missing at random, multiple imputations will be used. Sensitivity analyses will compare complete cases with imputed datasets. A two-sided alpha level of 0.05 will be considered statistically significant. For multiple secondary outcomes, false discovery rate (FDR) or Bonferroni correction may be applied. The results will be presented with appropriate tables and figures, for reproducibility.

A major aim of the monitoring and evaluation is to collect data to inform and support the planned longer-term roll-out of the program. Process evaluation will, therefore, be a major component of the evaluation plan, as well as using statistical methods for analyzing primary and secondary outcomes and the reference to where other details of the statistical analysis plan can be found, if not in the protocol.

An assessment of the costs of managing dental caries will be obtained through the triangulation of information collected from the National Insurance House and cost data sourced online. Given the variation in the provision of subsidized care across Romanian regions and the scarcity of information on public sector health care costs, private treatment costs are used as a proxy to estimate the direct costs of dental caries.

## 4. Expected Outcomes

The primary outcome of the trial will be the assessment of caries incidence, defined as the change in status according to the ICDAS index within the age groups. In addition, data analysis from the trial will provide information on children’s self-assessment of oral health practices and promote oral health activities for children with a focus on age-appropriate key messages and practical preparation for the implementation of the nursery intervention, which consists of a toothpaste/toothbrush scheme with free daily tooth brushing for every 4–6-year-old child attending the nursery. Depending on the region, by using the County Development Index, differences between regions will be assessed in order to establish the efficacy of the program at the national level.

Secondary outcomes will be the skills gained by dental hygienists to deliver oral health promotion to nursery nurses after specific training, and a comprehensive process of parental information and consent and validation that shapes healthy behaviours throughout life.

The clinical relevance of the findings is that preventive care includes good oral health behaviors such as low sugar consumption, health education, and daily oral hygiene using a toothbrush and fluoride toothpaste, as well as aids such as interdental brushes, dental floss, or mouthwashes that can help remove plaque and disrupt biofilm. Given the fact that caries prevalence has proven to be very high in Romanian children, the use of fluoridated toothpaste as a daily activity in kindergarten settings could lead to the improvement of their oral health status. Fluoride toothpastes play a dual role in caries prevention/management: firstly, by incorporating fluoride ions into demineralized enamel, replacing the missing hydroxyl component, and thus remineralization for incipient carious lesions [19,20,21,22], and secondly by reducing the activity of cariogenic bacteria like streptococci mutans [23,24,25,26] along with other antimicrobials. Several studies showed that daily tooth brushing with fluoridated toothpaste containing 1000–1500 ppm fluoride can reduce the biofilm by up to 42% and reverse existing incipient caries [21,27,28]. Daily tooth brushing is, therefore, very important to stop incipient caries and prevent the development of new lesions. At the same time, the intervention could create healthy behaviors at this very young age and, combined with oral health education this can lead to a healthy behavioral pattern throughout the whole life.

The clinical relevance of the chosen outcome efficacy and harm outcomes is strongly recommended.

## 5. Discussion

Dentistry is undergoing a major paradigm shift from a surgical specialty to a medical specialty; therefore, non-operative treatment plans will become more and more central, and this project aims to add a step to the pyramid of evidence. The significance of this intervention represents an innovative approach of implementing daily tooth brushing, using fluoridated toothpaste, as part of a supervised, standardized, and routinely delivered public health strategy, as a protective measure to prevent new carious lesions, as well as for its therapeutic potential under real-life conditions.

Although the preventive and therapeutic effects of fluoridated toothpaste have been extensively studied, the specific research gap addressed by this study lies in the fact that it is used within a school-based oral health intervention targeting high-risk young children.

By integrating fluoride exposure through supervised tooth brushing into the daily routine of children in an institutional setting, this model offers a potentially more effective and equitable method of improving oral health, particularly in populations where access to regular dental care and parental supervision at home may be limited.

These characteristics are important taking into account the diverse socio-economic settings, with low access to professional dental care, where cost-effective, self-administered interventions like fluoridated toothpaste can play a crucial role in improving oral health status.

Currently, oral health care for young children is insufficient. For this reason, it is important to create new medical teams and provide comprehensive training for dental hygienists. Dental hygienists play a very important role in oral health promotion and disease prevention, being in close contact with patients in clinical and community settings. They are able to provide education and early detection of oral diseases, deliver preventive measures, and create individual oral care plans. By having close relationships with patients, starting from very young ages, dental hygienists are able to influence long-term health behaviors and ensure consistent dental check-ups. Their role is even more important in preventive interventions for children in underprivileged communities, thereby reducing oral health inequalities and enhancing overall health status. Therefore, dental hygienists are of great importance in sustaining oral health but can also help to increase the importance of prevention within the health care systems. Until recently, Romania has lacked baseline data regarding the oral health status of children. A contemporary assessment is therefore mandatory in order to advance scientific understanding and allow for the development of adequate public health policies. During 2019–2020, the National Oral Health Survey evaluated a significant sample of children aged 5, 6, and 12 years, gathering baseline data on oral health according to the WHO guidelines. The data were used to estimate the distribution and severity of dental caries, the need for community-oriented disease prevention and health promotion, and the nature and urgency of the oral health interventions required. The survey also established how younger age groups can be reached and evaluated. Data show that only 14% of 6-year-old children have dmft (decayed-missing-filled teeth) 0 and the SiC index (significant caries index) of the same sample is 9.83. The high prevalence and severity of dental caries, significant oral health inequalities, and poor utilization of and access to services highlighted the need for a child oral health program. These findings are common to many other countries in the region, as well as other developing regions. Oral health status is strictly related to common risk factors, such as socio-economic status, education, and access and utilization of health care services [29,30,31,32,33]. Low socio-economic status leads to a decreased use of dental services, influenced by a lack of resources, treatment costs, and access to health care [30,34,35,36,37]. This is of great importance mainly in developing countries due to financial constraints [38], where discrepancies between high- and low-income families can lead to inequalities in dental care. Thus, children from low-income families tend to seek dental care mainly for curative and emergency treatment and usually do not benefit from health promotion or preventive care [4].

The aim of this intervention is to direct the traditional curative approach, which is essentially pathogenic, towards a preventive and promotional approach, with risk identification for timely, comprehensive, and inclusive care, involving all stakeholders, in order to contribute to the improvement of the oral health of the population, with a positive impact on general health.

This intervention could represent an example of good practice for the countries in South-East Europe. Starting from downstream oral health interventions, such as clinical prevention and oral health promotion which can improve the quality of children’s lives, the aim is to further develop the program towards upstream interventions [39,40,41]. The results from the trial will be further published in scientific journals and presented at appropriate conferences and will also be reported to the national stakeholders, governmental entities, and local authorities to further develop and promote the intervention.

The program also involves a novel curriculum for dental hygienists aiming to promote efficient workforce models; create primary care teams, including community health workers, in prevention and control; and build a supportive framework for the integration of caries prevention and control into overall health initiatives. The development of such a curriculum will be based on the dissemination of results from the evaluation of participating dental hygienist students.

Preventive care involves good oral health behaviors such as low sugar consumption, health education, and daily oral hygiene using a toothbrush and fluoridated toothpaste as well as auxiliary devices such as interdental brushes, dental floss, or mouthwashes which can help remove dental plaque and disrupt biofilm. Fluoride toothpastes can decrease the solubility of enamel and help remineralize incipient carious lesions [19,20,21,22]. In combination with other antimicrobial substances, it can also reduce the activity of cariogenic bacteria like streptococci mutans [23,24,25,26]. Research studies have shown the potential of fluoridated toothpaste to reverse incipient caries lesions and reduce the oral biofilm [21,27,28]. Therefore, daily tooth brushing is very important to arrest incipient caries lesions and prevent new lesions from appearing. Corroborating oral health education, this can lead to a healthy behavioral pattern throughout the whole life.

The role of fluoride has been often discussed in dental public health debates for many years, with scientists supporting its benefits for caries prevention but acknowledging also the need for appropriate use. Fluoride is recognized as the most effective measure to prevent dental caries by improving enamel resistance to acid attack and promoting the remineralization of early lesions [42]. Regular exposure to fluoride at therapeutic levels, from topical sources such as toothpaste or systemically by fluoridated water, has proven to significantly reduce caries risk across communities. However, there still are discussions regarding the method and level of fluoride delivery. While topical sources of fluoride contained in toothpastes are accepted and recommended, systemic fluoridation continues to be a controversial topic in some communities because of the risk of overexposure [43]. There are opinions that support local applications of fluoride, especially in areas where naturally occurring fluoride levels in water or soil may already be high, as excessive exposure can lead to dental fluorosis [44,45].

Moreover, there is a growing recognition of the importance of equitable access to fluoride-based prevention. Children from lower socio-economic backgrounds are often less likely to use fluoridated toothpaste or receive professional fluoride treatments, contributing to persistent oral health disparities [46]. As such, discussions now emphasize not just the benefits of fluoride itself, but also the importance of integrating fluoride use into broader oral health promotion strategies, education campaigns, and community outreach efforts, particularly in underserved populations [47].

Thus, fluoride remains a cornerstone of modern preventive dentistry, but its use should be tailored to individual and community needs, guided by up-to-date evidence, and delivered as part of a comprehensive oral health approach.

To assess the outcomes of this intervention, strengths and weaknesses have to be considered. The evaluation of the program consolidates and builds upon previous evaluation work of the National Oral Health Survey for children. Due to the fact that it is a pilot program aiming to be further implemented at a national level, one strong point is an evolving model in evaluation. This allows the evaluation to be responsive to issues emerging from its implementation and develops the program as a result of the evaluation findings.

The methods proposed for evaluation must be formative—to improve the program—and summative—to assess its impact. Evaluation activities include the collection of routine monitoring data linked to national datasets from the previous survey, the assessment of the impact of the program at different time intervals, and the assessment of economic outcomes.

The research team proposed to incorporate sustainability into the process taking into account the fact that health interventions often include a number of unquantifiable variables, which add a layer of complexity in terms of environmental appraisals. As a weak point, the discrepancy between the existence of evidence-based health promotion interventions and their use in practice is present in almost all medical fields but has been widely recognized as a challenge in dentistry. Clearly, the traditional ways of spreading information through scientific publication channels and passive instruction are not sufficient to reliably initiate and sustain new practices. To move evidence-based approaches into practice, more careful examinations of methods to introduce and sustain effective oral health practices are needed. For this project, we suggested a multi-staged framework approach. This resource is designed to interweave with the many other points of the project that have been developed to guide oral health professionals and dental organizations.

The obtained data from this trial will contribute to raising awareness among policy makers and health care professionals on the importance of oral health interventions at young ages and outline the care pathway and practical preparation for delivering interventions in the nurseries.

## 6. Dissemination of Findings

The results of the trial will be communicated to the parents of participating children through an individualized report of the data obtained after each clinical visit. The results will be disseminated through national impact workshops, publication of relevant results in peer-reviewed scientific journals, and a best practice guide at the end of the study.

## Figures and Tables

**Table 1:** Schematic diagram of the participant timeline.

	Study Period						
	Enrolment	Allocation	Post-Allocation				Close-Out
TIMEPOINT	−T1	0	T1	T2	T3	T4	T5
ENROLLMENT							
Eligibility screen	X						
Informed consent	X						
Allocation		X					
INTERVENTIONS							
Daily tooth-brushing scheme			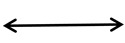				
Oral health education for children/caretakers			X	X	X	X	
Training for nursery staff			X		X		
Training for dental hygienists			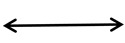				
ASSESSMENTS							
Self-assessment			X	X	X	X	
Clinical outcomes			X	X	X	X	X
Quantitative evaluation of nursery staff			X				
Quantitative evaluation of dental hygienists			X				
Qualitative evaluation of nursery staff				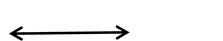			
Qualitative evaluation of dental hygienists				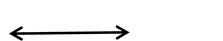			

## Data Availability

Access to the final trial dataset will be granted to the coordinator. The partner institutions will have access to the data that was collected by each of them.

## Abbreviations

The following abbreviations are used in this manuscript:

ECC	Early Childhood Caries
WHO	World Health Organization
ICDAS	International Caries Detection and Assessment System
ICCMS	International Caries Classification and Management System
SD	standard deviation
PP	Per Protocol
ITT	Intention to Treat
RR	risk ratio
NNT	number needed to treat
HR	hazard ratio
DMC	Data Monitoring Committee
dmft	decayed-missing-filled teeth
SiC	significant caries index
